# 

**Published:** 1955-09

**Authors:** 


MEDICAL JOURNAL OF THE SOUTH-WEST

*(Incorporating the Bristol Medico-Chirurgical Journal)*

Established 1883

VOL. 70 (v) September, 1955 NO. 257

### *Contents*

*PUBLISHED QUARTERLY*

*ANNUAL SUBSCRIPTION 16/- POST FREE*

PUBLISHED UNDER THE AUSPICES OF THE
BRISTOL MEDICO-CHIRURGICAL SOCIETY

### *Editorial Committee*

Dr. J. E. Cates, Department of Medicine, Bristol Royal Infirmary. *Editor.*
Dr. A. H. Gale, The University, Bristol 8. *Assistant Editor.*
Dr. J. Macrae, Ham Green Hospital. *Treasurer.*
Dr. N. J. Brown, Southmead Hospital, Bristol.
Mr. J. P. Partridge, Southmead Hospital, Bristol.

*Local Correspondents*

*Bath* . . Mr. A. D. Bateman, 3 The Circus, Bath.
*Exeter* . . Dr. L. N. Jackson, Mount Jocelyn, Crediton, Devon.
*Gloucester* . Dr. R. F. Jarrett, 9 College Green, Gloucester.
*Plymouth* . Dr. C. R. Croft, Lexden, Hartley Av., Plymouth.
*Taunton* . Dr. M. McAlley, 1 The Crescent, Taunton.
*Torquay* . Dr. A. Robinson Thomas, 20 Devon Square, Newton Abbot, Devon.
*Truro* . . Dr. F. D. M. Hocking, St. Andrews, Perranporth, Cornwall.
*Yeovil* . . Mr. J. A. Vere Nicoll, Knapp House, Yeovil, Somerset.

### NOTICE TO CONTRIBUTORS

Original articles are invited, provided they have not been published elsewhere. Notes of forthcoming events, meetings held, degrees conferred, staff appointments, and other local news are welcomed. The editorial committee reserves the right to make literary corrections.

Illustrations should always be supplied ready for reproduction. All re-touchings or other operations required will be charged to the author. Line drawings should be drawn in Indian ink. We regret that we must ask authors to pay for making blocks, if this is necessary.

J. W. ARROWSMITH LTD., WINTERSTOKE ROAD,
BRISTOL.

### *Notes and News*

The usual Sunday morning lecture-demonstrations for general practitioners were held from 27th March to 22nd May, 1955.

Intensive week-end courses for general practitioners were held at Exeter from 13th to 15th May, 1955, and at Cheltenham from 24th to 26th June, 1955.

### UNIVERSITY OF BRISTOL

Dr. R. F. Woolmer, promoted to a Readership in Anaesthetics.

Dr. T. D. Williams, B.SC., M.B., CH.B., Lecturer Grade II in Physiology, 25th March, 1955.

Dr. J. L. Jarvis, M.B., B.S., L.M.S.S.A., Dr. M. S. Dunnill, M.B., CH.B., Demonstrator in Pathology (2), 10th June, 1955.

Dr. T. M. Abbas, M.B., CH.B., M.D., F.R.C.S., M.R.C.O.G., Lecturer in Obstetrics and Gynaecology, 3rd May, 1955.

*DOCTOR OF MEDICINE*: Herford, M. E. M., and Schnieden, H.

*MASTER OF SURGERY*: Slade, P. R.

### *M.B , Ch.B. (JUNE,* 1955)

WITH SECOND CLASS HONOURS

Burge, Elizabeth—with distinction in Public Health.
Moore, Joyce, Esme—with distinction in Public Health.
Polkinghorne, Mavis Mary—with distinction in Medicine.

PASS

Allwood, Geoffrey Keith

Bate, Dorothy Frances

Bebell, Jean Margaret

Beckingham, Prudence Mary

Bolton, Hubert Fairfield

Charleston, Mervyn Threfall

Chesterfield, Margaret Edith

Clarke, John Picton James

Craven, Shirley Ada

Faireman, Edith

Farrelly, Francis Gerald

Foley, David John

Hartwell, James Keith

James, Andrew John Seldon

Jorro, Mary Theresa Catherine Ann

Lambert, Peter Michael

Laurence, Pamela Marjorie

Leighton, Penelope Anne

Mercer, Nancy—*with distinction in Public Health*

Midwinter, Ralph Edward

Naylor, Gerald Alexander

Neilson, Jeffrey Benjamin—*with distinction in Public Health*

Nicholls, Hedley John Leslie

Roylance, Peter James

Simpson, Donald Watson—*with distinction in Obstetrics and Gynaecology*

Smith, Edward Michael

Stewart, Anne Lindsay

Wallace, Edgar

*In Group I completing the examination*

Keevil, Janet Hazel
McCormick, Robert George

*Francis Dudley Memorial Prize for* 1954: C. C. Entwistle.

*The Crosby Leonard Prize for the Present Session*: A. Morgan.

*The Dental Gold Medal for* 1954: M. D. H. Allen.

*The Thomas Francis Edgeworth and Francis Henry Edgeworth Prize in Physiology*: P. R. Golding.

*Mackie Pathology Prize for* 1955: J. H. Williams.

*Hewer Pathology Prize for* 1954-55: J. A. Hayes.

*Tibbits Memorial Prize for* 1955: Penelope A. Leighton.

*F.R.C.S.*: Walder, D. N.

*M.R.C.O.G.*: Soltau, D. H. K.

*D.M.R.D.*: Moore, E. W.

*D.P.M.*: (*Part* 2) *Conjoint:* Galla, T., Joyce, M. H. B., Seager, C. P. and Walker, D. L.

*D.P.H.*: O'Dea, Margaret M. J., Sefton, Elizabeth M., Speller, P. J. and Reilly, M. D.

*D.A.*: Forrest, Mari, Christie, Anne W., Haines, Ann M.

*New Year Honour*—1955: A. F. Alford, M.B., CH.B., C.B.E. (Civil Division).

### *Appointments*

### *UNITED BRISTOL HOSPITALS*

| *Name* | *Appointment* | *Formerly* |
|---|---|---|
| CHITTY, K., M.B., B.CH., F.R.C.S. | Senior Registrar in General Surgery, United Bristol Hospitals. | Senior Registrar in Genito-Urinary Surgery, United Bristol Hospitals |
| AKEHURST, A. C., M.B., B.S., F.R.C.S. | Registrar in General Surgery, United Bristol Hospitals. | Senior House Officer in General Surgery, United Bristol Hospitals. |
| MCINNES, A. D., M.B., CH.B., M.R.C.P.E. | Senior Registrar in General Medicine, United Bristol Hospitals. | Registrar in Medicine, Western Infirmary, Glasgow. |
| EDWARDS, E. M., M.B., B.S., M.R.C.O.G. | Senior Registrar in Obstetrics & Gynaecology, Southmead Hospital. | Registrar in Obstetrics & Gynaecology, United Bristol Hospitals. |
| TREISMAN, O. S., M.B., B.CH., M.R.C.O.G. | Registrar in Obstetrics & Gynaecology, United Bristol Hospitals. | Senior Medical Officer in Obstetrics & Gynaecology, King Edward VII Hospital, Durban. |
| RICHARD, D. R., B.M., B.CH. | Registrar in Orthopaedics, United Bristol Hospitals. | Registrar in Surgery, Weston-super-Mare General Hospital. |
| HARDY, H. M., M.B., CH.B., D.A. | Senior Registrar in Anaesthetics, Leeds. | Registrar in Anaesthetics, United Bristol Hospitals. |
| CHANDLER, C. C. D., M.R.C.S., L.R.C.P., D.A. | Registrar in Anaesthetics, United Bristol Hospitals. | Registrar in Anaesthetics, Kingston Hospital. |
| SIMPSON, K., M.D., B.S., M.R.C.P. | Registrar in Paediatrics, United Bristol Hospitals. | Registrar to Children's Department, St. Thomas' Hospital. |
| COLE, MISS P. M., M.B., B.S., D.C.H. | Registrar in Paediatrics, United Bristol Hospitals. | Registrar in Paediatrics, Plymouth. |
| MCCORMICK, A. J. A., M.B., CH.B., D.O.M.S., F.R.C.S. | Registrar in Ophthalmology, United Bristol Hospitals. | Senior Registrar to Eye Department, Edinburgh Royal Infirmary. |
| HOLT, J. A. G., M.B., CH.B., F.R.C.S., D.OBST.R.C.O.G. | Registrar in Radiotherapy, United Bristol Hospitals. | Senior House Officer in Radiotherapy, United Bristol Hospitals. |
| SLADE, N., M.B., CH.B., F.R.C.S. | Senior Registrar in Genito-Urinary Surgery, United Bristol Hospitals. | Surgeon, Port Sudan Civil Hospital. |
| ST. CLAIR, J. D., M.B., CH.B., D.A., F.F.A.R.C.S. | Registrar in Anaesthetics, United Bristol Hospitals. | National Service, R.A.M.C. |
| EVANS, W. A., M.B., CH.B. | Registrar in Radio-diagnosis (part-time), United Bristol Hospitals. | Radiologist, Grey Hospital, Greymouth, New Zealand. |

### *SOUTH-WESTERN REGIONAL HOSPITAL BOARD*

*September* 1954 *to June* 1955

### *BRISTOL*

| *Name* | *Appointment* | *Formerly* |
|---|---|---|
| WALKER, W. LUMSDEN, M.B., CH.B., D.P.H., D.P.M. | Medical Superintendent, Hortham-Brentry Hospitals Group. | Senior Registrar in Mental Deficiency & Child Psychiatry, Bristol. |
| MAKSIMCZYK, K., M.B., CH.B. (POLISH SCHOOL OF MEDICINE, EDINBURGH). | Asst. Medical Director, Mass Radiography Units, Bristol (S.H.M.O.). | Asst. Medical Director, Mass Radiography Units, Edinburgh. |
| WALLEY, R. V., B.A., M.B., B.CHIR. | Deputy Resident Physician (S.H.M.O.), Ham Green Hospital, near Bristol. | J.H.M.O., Ham Green Hospital. |
| PARTRIDGE, J. P., M.B., B.S., F.R.C.S. | Senior Registrar in General Surgery, Southmead Hospital. | Senior Registrar in General Surgery, United Bristol Hospitals. |
| WATERFALL, JOAN M., M.B., CH.B., D.A., PRIMARY F.F.A., R.C.S. | Temporary Senior Anaesthetic Registrar, Frenchay Hospital. | Locum Senior Anaesthetic Registrar, The London Hospital. |
| WEST, MARY F., M.B., B.S.-(LOND), D.A. (PART I). | Anaesthetic Registrar, Southmead Hospital, Bristol. | Locum S.H.O. Anaesthetist, Swansea General Hospital. |
| FLETCHER, J. P., B.D.S., L.D.S., R.C.S., PRIMARY F.D.S., R.C.S. | Registrar in Dental Surgery, Bristol. | National Service in the Army. |
| WELLS, A. MARO, M.B., CH.B., M.R.C.S., L.R.C.P., D.OBST., R.C.O.G., D.C.H. | Medical Registrar, Southmead Hospital, Bristol. | S.R.O., Bristol Royal Infirmary, & S.H.O. to Professor Perry & Dr. Cates. |
| HARGADON, E. J., M.B., B.CH., M.R.C.S., L.R.C.P. PRIMARY F.R.C.S. | Surgical Registrar, Cossham-Frenchay Hospitals Group, Bristol. | Surgical Registrar, Caerphilly & District Miners' Hospital. |
| LEAHY, T. C., M.B., B.CH., B.A.O. | S.H.M.O., Hortham-Brentry Hospitals Group. | Locum S.H.M.O., Hortham-Brentry Hospitals Group. |
| BRADFIELD, A. M., M.B., CH.B., (NEW ZEALAND). | Psychiatric Registrar, Bristol Mental Hospitals. | Senior House Physician, St. Benedict's Hospital, Tooting. |
| GALLA, T., M.B., CH.B., D.P.M. | Senior Psychiatric Registrar, Bristol Mental Hospitals. | Psychiatric Registrar, Bristol Mental Hospitals. |
| LEIGH, H. I., M.B., CH.B., | Registrar in the Dept. of Neurological Surgery, Frenchay Hospital, Bristol. | Medical Officer, R.A.F. |
| EDWARDS, E. M., M.R.C.S., L.R.C.P., M.B., B.S., D.OBST.R.C.O.G., M.R.C.O.G. | Temporary Senior Registrar in Obstetrics & Gynaecology, Southmead Hospital, Bristol. | Registrar in Obstetrics & Gynaecology, United Bristol Hospitals. |
| JOLLY, G. F., M.R.C.S., L.R.C.P., M.A., M.B., B.CH., D.OBST.R.C.O.G., M.R.C.O.G. | Registrar in Obstetrics and Gynaecology, Southmead Hospital, Bristol. | Locum Registrar to the Professorial Unit, United Birmingham Hospitals. |
| STEWART, R. L. N., M.B., CH.B., D.O. | Opthalmologist, Bristol, (S.H.M.O.). | Senior Resident at Bristol Eye Hospital. |
| BROCK, B. H., M.R.C.S., L.R.C.P., M.B., B.CHIR., F.R.C.S. | Orthopaedic Registrar, United Bristol Hospitals and Winford Orthopaedic Hospital. | Orthopaedic Registrar, St. Olave's Hospital, Rotherhythe. |
| FULLER, ANGELA D., M.R.C.S., L.R.C.P., M.B., B.CHIR., D.C.H. | Paediatric Registrar, Southmead Hospital, Bristol. | R.M.O., Infectious Diseases Unit, Southampton Chest Hospital. |
| GORDON, A., M.B., CH.B. | Registrar to the Department of Pathology, Frenchay Hospital, Bristol. | S.H.O. to the Department of Pathology, Frenchay Hospital. |
| POLLARD, B. R., M.R.C.S., L.R.C.P., M.B., B.CHIR. | Assistant Pathologist, Frenchay Hospital, Bristol. | Senior Registrar to Department of Clinical Bacteriology, Wright-Fleming Institute, St. Mary's Hospital. |
| DOYLE, F. H., B.SC., M.B., CH.B. | Part-time Registrar in Radiodiagnosis, Bristol Clinical Area. | S.H.O. in General Surgery, Maryfield Hospital, Dundee. |
| SNELL, F. W. G., M.B., CH.B. | Part-time Registrar in Radiodiagnosis, Bristol Clinical Area. | Surgical Registrar, Jersey General Hospital. |

### *NORTH GLOUCESTERSHIRE*

| *Name* | *Appointment* | *Formerly* |
|---|---|---|
| FEARNLEY, G. R., M.R.C.S., L.R.C.P., M.B., B.S., M.R.C.P., M.D. | Consultant Physician, North Gloucestershire Clinical Area. | Lecturer in Medicine, Postgraduate Medical School of London, and Hon. Consultant Physician, Hammersmith Hospital. |
| THEOBALDS, J. R., M.R.C.S. L.R.C.P. | Medical Registrar, Cheltenham General Eye & Children's Hospital. | Medical Registrar, Royal Salop Infirmary, Shrewsbury. |
| VILLET, D. DE G., M.B., CH.B., (CAPE TOWN), F.R.C.S. | Surgical Registrar, Gloucestershire Royal Hospital, Gloucester. | Locum Surgical Registrar, Lambeth Hospital. |
| HAROON, S., M.B., B.S. (PUNJAB), L.M., D.G.O., | Registrar in Obstetrics and Gynaecology, Gloucestershire Royal Hospital, Gloucester. | Gynaecological House Surgeon, City General Hospital, Gloucester. |

### *BATH*

| *Name* | *Appointment* | *Formerly* |
|---|---|---|
| SCHOFIELD, T. L., M.B., M.R.C.S., L.R.C.P., F.R.C.S., CH.M. | Consultant Surgeon, Bath, Clinical Area. | Lecturer in Surgery & 1st Asst. to the Professor of Surgery, Liverpool University. Hon. Consultant Surgeon, Broadgreen Hospital, Liverpool. |
| JONES, E. R., M.R.C.S., L.R.C.P., D.P.M. | Assistant Psychiatrist, Mendip Hospital, Wells. | Assistant Psychiatrist, Cefn Coed Hospital, Swansea. |
| KNUTZEN, J. S., M.B., CH.B., (CAPE TOWN). | E.N.T. Registrar, Bath Group of Hospitals. | House Surgeon, Royal United Hospital, Bath. |
| SAXTON, H. M., M.B., B.S. | Medical Registrar, St. Martins Hospital and Royal National Hospitals for Rheumatic Diseases, Bath. | Medical Superintendent, St. Mary's Hospital, London. |
| HIBBARD, B. M., M.B., B.S., M.R.C.S., L.R.C.P., D.OBST.-R.C.O.G. | Registrar in Obstetrics and Gynaecology, Bath Group of Hospitals. | House Physician, Royal Northern Hospital, London. |
| NEATBY, G. M., M.R.C.S., L.R.C.P., D.O.M.S., B.A., M.A. | Clinical Asst. in Ophthalmology, Bath Eye Infirmary—one session in the S.H.M.O. grade. | Is in Ophthalmic practice, Bristol. |
| TUPMAN, G. S., B.SC., M.B., CH.B., F.R.C.S. | Senior Orthopaedic Registrar, Bath Clinical Area. | Orthopaedic Registrar, Lord Mayor Treloar Orthopaedic Hospital, Alton. |

### *SOUTH SOMERSET*

| *Name* | *Appointment* | *Formerly* |
|---|---|---|
| NELSON, M. P., B.A., M.R.C.S., L.R.C.P., D.P.M. | Medical Superintendent, Sandhill Park Hospital Group, Bishops Lydeard. | S.H.M.O., Harperbury Hospital near St. Albans. |
| HUBBARD, ST. C. M. L'A., M.R.C.S., L.R.C.P., M.B., B.S. | Clinical Assistant in Orthopaedic & Traumatic Surgery, Taunton & Somerset Hospital. (1 session). | Is in general practice in Taunton. |

### *DEVON AND CORNWALL*

| *Name* | *Appointment* | *Formerly* |
|---|---|---|
| DAWSON, J. J. Y., M.R.C.S., L.R.C.P., M.B., B.S., M.D., M.R.C.P. | Senior Consultant Chest Physician, Plymouth Clinical Area. | Medical Chief Assistant, Brompton Hospital. |
| EARLE, B. V., M.B., B.CH., B.A.O., M.R.C.P., M.D., D.P.M. | Consultant Psychiatrist, Exeter Clinical Area. | Locum Consultant Psychiatrist, Powick Hospital, Worcester. |
| MOORE, H. D., M.B., B.S., (SYDNEY), F.R.C.S., | Consultant Surgeon, Exeter Clinical Area. | Senior Surgical Tutor, University of Leeds. |
| MOYLE, I. R., M.A., M.R.C.S., L.R.C.P., D.M.R.D. | Consultant in Radio-diagnosis, Plymouth Clinical Area. | Registrar, Dept. of Radiodiagnosis, United Bristol Hospitals. |
| MACPHERSON, J., M.B., CH.B., F.R.C.S., M.R.C.O.G. | Consultant Obstetrician and Gynaecologist, Torquay. | Senior Registrar in Obstetrics & Gynaecology, Mill Road, Maternity Hospital, Liverpool. |

*DEVON AND CORNWALL*—continued.

| *Name* | *Appointment* | *Formerly* |
|---|---|---|
| PAYNE, I. W., M.B., CH.B., D.O.M.S., F.R.C.S. | Consultant Ophthalmologist, Plymouth Clinical Area. | Senior Registrar, Royal Eye Hospital, London, S.E.1. |
| STORMONT, H. A. W., M.R.C.S., L.R.C.P. | General Practitioner Anaesthetist, Ilfracombe & Dist. Hospital. (1 session). | Is in general practice in Ilfracombe. |
| WINFIELD, A. G., M.R.C.S., L.R.C.P., M.B., B.S. | General Practitioner Anaesthetist, Tavistock Hospital. (1 session). | Is in general practice in Tavistock. |
| ISAAC, D. H., M.R.C.S., L.R.C.P., M.B., B.S., M.D., M.R.C.P. | Senior Medical Registrar, South Devon & E. Cornwall Hospital, Plymouth (Joint appt. with the United Bristol Hospitals). | Medical 1st Assistant, Sheffield Royal Infirmary. |
| PYBUS, P. K., M.B., B.CHIR., M.R.C.S., L.R.C.P., D.OBST.-R.C.O.G., PRIMARY F.R.C.S. | Surgical Registrar, Torquay and Newton Abbot Hospitals. | Locum Surgical Registrar, Poplar Hospital, London. |
| STRANGE, D. M., M.A., L.S.A. | S.H.M.O., Royal Western Counties Institution, Starcross. | J.H.M.O., Mendip Hospital, Wells. |
| ROBERTS, A. G., B.SC., M.B., B.CH. | Psychiatric Registrar, Moorhaven Hospital, Ivybridge. | S.H.O. in Psychiatry, Whitchurch Hospital, Cardiff. |
| REILLY, M. C. T., M.B., B.S., M.R.C.S., L.R.C.P., F.R.C.S., M.S. | Consultant Surgeon, Plymouth Clinical Area. | Surgical 1st Assistant, The London Hospital. |
| GALLAGHER, M. J., M.B., B.S. (QUEENSLAND), F.R.C.S. | Orthopaedic Registrar, Princess Elizabeth Orthopaedic Hospital, Exeter. | S.H.O. Princess Elizabeth Orthopaedic Hospital. |
| COLE, PAULINE, M.B., B.S., D.C.H. | Registrars in Paediatrics to the South Devon and East Cornwall Hospital, Plymouth, and the United Bristol Hospitals (interchange appointments). | Registrar in Paediatrics, Queen Elizabeth Hospital for Children, Hackney. |
| LLOYD, JUNE K., M.B., CH.B, M.R.C.P. | | S.H.O. in General Medicine, United Bristol Hospitals. |
| CHUDECKI, B., M.B., CH.B., (CRACOW), D.M.R.T. | Assistant Radiotherapist, Exeter Clinical Area. | Registrar to Radiotherapy Dept. Charing Cross Hospital. |
| MAINPRISE, J. V., M.B., CH.B. | Assistant Venereologist, Torquay. (2 sessions). | Undertaking same post on locum basis. |

*ELSEWHERE*

| *Name* | *Appointment* | *Formerly* |
|---|---|---|
| HAWKINS, J. R., M.B., D.P.M. | Consultant Psychiatrist, Winterton Hospital, Sedgefield, Co. Durham. | — |
| FRANCIS, M. J., M.B., D.C.H., D.A. | Junior Resident Anaesthetist, Hospital for Sick Children, Gt. Ormond St., London, W.C.1. | — |
| BOSE, S. N., D.A. | Senior Lecturer in Anaesthetics, Kitchener School of Medicine, Khartoum. | — |
| PERSEY, DOROTHY J., M.B. BRISTOL, D.C.H. | Asst. Maternity and Child Welfare M.O. and School M.O., Derbyshire. | — |
| LAURANCE, B. M., M.B., B.S., M.R.C.P., D.C.H. | Consultant Paediatrician, Sheffield Regional Hospital Board. | Senior Registrar in Paediatrics, United Bristol Hospitals. |
| LEATHER, H. M., M.B., B.CH., M.R.C.P. | First Assistant in Medicine, Makerere College, Uganda. | Senior Registrar in General Medicine, United Bristol Hospitals. |
| FREEMAN, R. H., M.B., CH.B., F.R.C.S. | Senior Registrar in Orthopaedics, King's College Hospital. | Registrar in Orthopaedics, United Bristol Hospitals. |
| MARCH, H. F., M.B., B.S., M.R.C.P., D.M.R.D. | Consultant Radiologist, Leeds Regional Hospital Board. | Senior Registrar in Radiodiagnosis, United Bristol Hospitals. |

